# Temporal Relationship of Acute Rheumatic Fever Following COVID-19 Infection: A Pediatric Case Report

**DOI:** 10.7759/cureus.50147

**Published:** 2023-12-07

**Authors:** Mohammed A Alkhamis, Hussain J Aljubran, Maitham J Aljubran, Ahmed H Buzaid, Mariam A Alali, Maryam H Alessa, Abdullah H Almeshari

**Affiliations:** 1 Pediatric Department, Maternity and Children Hospital, Al-Ahsa, SAU; 2 Medicine, Imam Abdulrahman Bin Faisal University, Dammam, SAU

**Keywords:** post-covid-19 condition, pediatric, acute rheumatic fever, covid-19, coronavirus

## Abstract

Coronavirus disease 2019 (COVID-19) has the potential to trigger the onset of autoimmune disorders, one of which is acute rheumatic fever (ARF). ARF is an immune system response that can manifest after an individual has been infected with *Streptococcus pyogenes. *In this study, we document a unique case involving a previously healthy child who exhibited symptoms of fever, polyarthritis, and ankle swelling after history of COVID-19 infection one month ago. This rare pediatric case report discussed the occurrence of ARF after a one-month period of COVID-19 infection, and we observed significant improvement in our patient after a three-month treatment regimen.

## Introduction

The World Health Organization declared the novel coronavirus disease 2019 (COVID-19) outbreak a pandemic on March 11, 2020. This declaration marked a significant recognition of the global spread and severity of the virus. It was the first time a coronavirus had caused a pandemic [1]. Most individuals infected with COVID-19 experience mild symptoms similar to those of flu. However, around 12.29% of patients become critically ill, with some developing various complications related to the virus [2]. This aspect of COVID-19 is a significant research topic, as it encompasses a range of outcomes. Notably, it is worth noting that bacterial co-infection alongside COVID-19 has been observed in pediatric populations [3].

Previous studies have reported clusters of pediatric patients who experienced systemic inflammatory disorders associated with COVID-19 exposure [4,5]. Although the mortality rate related to COVID-19 is extremely low, in the later phase of the infection, more serious conditions involving children have been identified, including acute rheumatic fever (ARF) [6-8]. The Jones diagnostic criteria summarize the symptoms of ARF, which is a condition that affects the whole body and is linked to *Streptococcus pyogenes* (group A streptococcus [GAS]) infection. Patients with ARF typically present with choreiform movements, migrating arthralgias, skin problems, and cardiac issues [9].

Currently, extensive data regarding the potential relationship between ARF and COVID-19 are lacking. The number of reported cases of individuals with both conditions is limited [8,10]. Herein, we report on a pediatric patient presenting with ARF after one month of COVID-19 infection.

## Case presentation

A previously healthy 13-year-old boy presented to our pediatric emergency department (PED) complaining chiefly of fever, joint pain, and swelling involving both the right hand and left ankle. The fever had started one day before, swelling started in the right hand one week previously, and two days after that, the left ankle began swelling. The patient had no cough, shortness of breath, vomiting, diarrhea, or history of trauma. His family history of rhematic heart disease and autoimmune disease was negative. However, the patient had a history of a positive COVID-19 infection one month ago, with cough and runny nose as the initial presentation and he has not been hospitalized during that period. The COVID-19 serology was performed twice as it was positive at the initial presentation of COVID-19, while it was negative when the patient presented with ARF.

Upon arrival at the PED, the patient looked well and was alert, active, afebrile, and had stable vital signs. Additionally, a pansystolic murmur, mainly at the apex and radiating to the axilla, was detected during the cardiovascular examination. The musculoskeletal examination detected swelling and a decreased range of motion in both joints without tenderness or redness.

The initial laboratory tests performed in the PED were significant for elevated inflammatory markers (erythrocyte sedimentation rate [ESR]: 110 mm/1st hr; C-reactive protein [CRP]: 48 mg/L), microcytic anemia (hemoglobin: 11.1 g/dl), hyperalbuminemia (albumin: 81.5 g/L), and a borderline antistreptolysin O (ASO) titer (200 IU/ml) (Table 1).

**Table 1:** Initial laboratory findings at the PED.

Tests	Reference range	Patient values
Complete blood count		
White blood cell (WBC)	5-15.5 x 10^3^	10.67
Hemoglobin	11.5-12.5 g/dl	11.1
Mean corpuscular volume (MCV)	80-100 fl	66.5
Platelet	150-350 x10^3 ^/ ml	401
Erythrocyte sedimentation rate (ESR)	0-10 mm/1^st^ hr	110
C-Reactive Protein (CRP)	8-10 mg/L	48
Serum biochemistry		
Blood urea nitrogen (BUN)	3.2-7.9 mmol/L	3.12
Serum creatinine	27.4-53.93 mmol/L	44.01
Sodium (Na)	135-145 mmol/L	135
Potassium (K)	3.4-4.7 mmol/L	4.1
Calcium (Ca)	2.3-2.6 mmol/L	2.36
Aspartate transaminase (AST)	21-44 U/L	14
Alanine transaminase (ALT)	9-25 U/L	12
Albumin	38-47 g/L	81.5
Total Protein	60-83 g/L	81.5
Lactate Dehydrogenase (LDH)	140-180 U/L	155
Creatine Kinase (CK)	55-170 U/L	74
Creatine Kinase MB (CK-MB)	5-25 IU/L	23
Antistreptolysin O Titer (ASO)	<200 IU/mL	200
Brucella titer	-	Negative

An electrocardiogram (ECG) was performed and showed a normal sinus rhythm with a normal axis and a QT interval of 0.39 seconds with a normal PR interval. The chest X-ray was normal, and both the COVID-19 swab and throat culture swab were negative. The joint pain and swelling rapidly responded to non-steroidal anti-inflammatory drug (NSAID). A transthoracic echocardiography (TTE) that was performed to assess the heart valves showed moderate mitral regurgitation with mild aortic regurgitation at his first presentation. The patient was diagnosed with ARF and started on an afterload reducing agent (enalapril 2.5 mg orally twice a day) and monthly benzathine penicillin (1.2 million units intramuscular every 28 days), in addition to naproxen 250 mg orally twice a day. After one week of the intiation of the treatment, the patient started to develop Sydenham chorea in the form of uncontrollable dance-like motions, which further support the diagnosis of ARF. The second TTE after around one and a half month of his first presentation showed improvement in the mitral and aortic regurgitation compared to the previous TTE with lower inflammatory markers compared to the first presentation (ESR = 41 mm/1st hr), so the treating physicians elected to continue the same management. Nine weeks from the beginning of the treatment, the third TTE was performed and showed moderate mitral regurgitation and no more aortic regurgitation (Video 1), and the inflammatory markers were normal, so enalapril and naproxen were stopped. The last TTE three months later showed complete resolution of the mitral and aortic regurgitation (Video 2). Since the patient had migratory arthritis that rapidly responded to NSAID and evidence of carditis, in addition to borderline ASO titer, we diagnosed this patient as ARF. Accordingly, the patient was kept on and monthly benzathine penicillin (1.2 million units intramuscular every 28 days) for a period of 10 years as a secondary prophylaxis.

**Video 1:** Four chambers view in transthoracic echocardiography (TTE) showing moderate mitral regurgitation.

**Video 2:** Four chambers view in transthoracic echocardiography (TTE) after three months of treatment showing resolution of mitral regurgitation.

## Discussion

ARF is a significant contributor to acquired heart disease among children. The incidence of ARF varies globally. In developed countries, the annual incidence is less than 0.5 cases per 100,000 people. However, in developing countries, the incidence can be as high as over 100 cases per 100,000 people [11]. Previous studies have reported a prevalence of 0.88% of ARF concurrent with COVID-19 infection [8,10]. However, none of these studies included a pediatric case of ARF with a one-month history of COVID-19 infection prior to presentation, like our case.

ARF is a major cause of acquired heart disease in pediatric patients that is caused by an autoimmune response to throat infection with GAS [12]. The diagnostic criteria for ARF are based on the Jones criteria, which were revised in 2015 [13]. The criteria include both major and minor manifestations of the disease. Risk stratification has also been introduced, categorizing populations into low-risk and moderate-to-high-risk groups. The major diagnostic criteria for ARF include carditis, arthritis, chorea, erythema marginatum, and subcutaneous nodules. The minor criteria include arthralgia, hyperpyrexia, high ESR levels, high CRP levels, and a prolonged PR interval [13]. Our patient satisfied the revised Jones criteria with a category of moderate-to-high risk. This was confirmed by the presence of polyarthritis, as well as TTE results that indicated the presence of carditis. It is crucial to start antibiotic therapy once ARF is confirmed [14]. Penicillin has been identified as the most effective therapy for eradicating GAS in the pharynx and is recommended as a secondary preventive measure in cases of ARF and rheumatic heart disease [15]. The treatment goals for ARF involve eliminating the GAS infection with antibiotics and addressing the clinical manifestations such as arthritis, carditis, and chorea [16]. For this purpose, a single dose of intramuscular benzathine penicillin is used to eradicate GAS carriage, followed by regular doses at three-week intervals for secondary prophylaxis for a period of 10 years. After three months of this treatment regimen, our patient improved and did not need any further intervention. Interestingly, the presence of a previous COVID-19 infection with previously positive COVID-19 serology did not affect the treatment plan.

To the best of our knowledge, there has been only one report of ARF in pediatric patients with coexisting COVID-19 infections [10]. In this report, we presented a pediatric case of ARF after one month of COVID-19 infection with a presentation totally different from those included in previous studies, which highlights the need for further investigation into the impact of COVID-19 on ARF. The exact relation between ARF and COVID-19 is unclear. Recent studies have shown that COVID-19 can lead to the development of autoimmune diseases in some individuals [17]. The immunogenic effects of the virus, such as molecular mimicry by viral proteins and tissue damage, may contribute to immune dysregulation and the onset of autoimmunity, which could explain why this patient presented with ARF after he had been infected with COVID-19 one month previously [17]. Moreover, the wide range of autoimmune conditions observed in COVID-19 patients highlights the need for further investigation into the molecular mechanisms underlying COVID-19-related immune dysregulation. Understanding these mechanisms could potentially lead to the early diagnosis and effective management of autoimmune diseases associated with COVID-19 [18].

## Conclusions

ARF is a prevalent form of acquired heart disease in children globally, particularly among those aged five to 14 years. It is believed to occur due to an autoimmune response triggered by molecular mimicry following a GAS infection. This autoimmune cross-reactivity leads to the development of symptoms associated with ARF. The relation between ARF and COVID-19 is not totally understood. There have been limited published cases concerning ARF following COVID-19. Therefore, this case report suggests that ARF can occur even after a one-month period of COVID-19.
